# Bioactive Peptides in Human Health and Disease

**DOI:** 10.3390/ijms24065837

**Published:** 2023-03-19

**Authors:** Serena Martini, Davide Tagliazucchi

**Affiliations:** Department of Life Sciences, University of Modena and Reggio Emilia, Via Amendola 2, 42100 Reggio Emilia, Italy; serena.martini@unimore.it

Bioactive peptides are defined as short amino acid sequences that may have specific physiological functions, ultimately affecting human health and protecting against the development of several diseases. There are numerous sources of bioactive peptides, and they can be roughly classified as endogenous and exogenous bioactive peptides.

Endogenous bioactive peptides are already present in the organism, where they are produced by specific organs, tissues or cells and are able to modulate physiological functions in the organism itself. Examples of endogenous bioactive peptides are peptide hormones (such as vasopressin, luteinizing hormone, vasoactive intestinal peptide, gastrin, adropin, etc.) that are involved in the control of several physiological functions which include, among others, the regulation of cardio-vascular, reproductive and gastro-intestinal systems, appetite, metabolism and energy homeostasis. Other important classes of endogenous bioactive peptides are represented by neuropeptides (such as enkephalin, substance P, oxytocin, etc.) that are involved in modulating the function of the nervous system, and anti-microbial peptides mainly produced by immune cells that play an important role in inflammation and in the defence against several microorganisms. Apart from their physiological role, endogenous bioactive peptides can have various therapeutic applications or be used as a template for the design of new bioactive peptides or pseudopeptides.

On the other hand, exogenous bioactive peptides are not naturally present in the body and are produced by protein hydrolysis or synthesis. The main source of exogenous bioactive peptides is food, especially dairy products such as milk, fermented milk or cheeses. Nevertheless, bioactive peptides have also been identified after the hydrolysis of proteins from meat, plant and marine foods. Several biological activities have been ascribed to exogenous bioactive peptides, such as anti-hypertensive, anti-diabetic, anti-inflammatory, anti-microbial, immunomodulatory, and opioid activities. Several exogenous bioactive peptides share structural features with endogenous bioactive peptides and may act through similar mechanisms or in synergy with endogenous bioactive peptides.

Therefore, it is not surprising that bioactive peptides represent a fervid and growing field of research in different areas such as medicine, nutrition and pharmacology.

This Special Issue collects ten articles (eight research articles and two review articles) encompassing several aspects of bioactive peptides in the prevention of some diseases related to their anti-cancer, anti-microbial, neuroprotective and cardio-vascular protective activities. 

Three published articles investigated the potential role of some peptides in cancer therapy. 

The paper by Liu et al. [1] reported the effectiveness of a newly identified peptide for the development of a T cell-based cancer immunotherapy. Glioblastoma tumour cells over-express the transcription factor SOX11, which is low-expressed in normal cells and has been identified as a tumour-associated antigen recognized by CD8^+^ T cells. In their study, Liu et al. [1] firstly performed an in silico screening of SOX11-derived peptides for their potential ability to bind the major histocompatibility complex (HLA-A*0201) involved in the recognition of the SOX11 antigen in glioblastoma by CD8^+^ T cells. They identified ten SOX11-derived peptides with a predicted ability to bind HLA-A*0201. Next, they found by in vitro binding assay that one peptide (FMACSPVAL) was a strong ligand of HLA-A*0201 in cultured T cells. They further demonstrated the ability of FMACSPVAL to generate SOX11-specific CD8+ T cells ex vivo. They concluded that FMACSPVAL can be a promising peptide for anti-cancer immunotherapy, thanks to its ability to bind and activate SOX11-specific CD8+ T cells.

In a second study, Zhang et al. [2] identified the binding receptor of a peptide that can be used for the targeted delivery of drugs in anti-cancer immunotherapy on the surface of monocytes/macrophages. The designed peptide (with sequence NWYLPWLGTNDW) was able to bind prohibitins on the cell surface of monocytes/macrophages. The development of monocytes/macrophages targeting peptides able to deliver therapeutics inside the cells is a promising strategy in anti-cancer immunotherapy. Macrophages are an important component of the tumour microenvironment, and the so-called tumour-associated macrophages are involved in the suppression of anti-tumour immunity and in the resistance to chemotherapies.

In an additional published article, Bhattarai and colleagues [3] developed an effective computational approach for the in silico prediction of the anti-cancer potential of specific peptides. The method allows the screening of numerous peptides for their anti-cancer potential, avoiding the time-consuming and expensive classical approach for bioactive peptides discovery.

The search for new bioactive peptides with anti-viral and anti-microbial properties is a very active and appealing field of research.

In their article, Fernandez-Fuentes et al. [4] designed, by a computational approach, a set of peptides able to target the SARS-CoV-2 spike receptor binding domain-ACE2 interface. These peptides may potentially limit the interaction between SARS-CoV-2 spike protein and its receptor on the cell surface (ACE2), decreasing the ability of the virus to infect cells. Despite the limitations of the in silico approach, this study may be useful to develop new therapeutic molecules and strategies to contrast SARS-CoV-2 infection.

In another study, Kotynia and co-workers [5] identified three novel anti-microbial pseudopeptides containing the non-proteinogenic amino acids ornithine and 2,4-diaminobutyric acid. These pseudopeptides are metal chelators able to interact with copper and nickel ions. In addition to their anti-microbial activity, the three peptides did not show any cytotoxic activity and presented additional biological activities such as anti-inflammatory activity and the ability to decrease the LPS-induced cytotoxicity, suggesting their potential application for the treatment of bacterial infections.

Perlikowska and collaborator [6] investigated the ability of known endogenous and exogenous peptides to prevent cell damages in an in vitro model of Parkinson’s disease. They used endogenous endomorphins 1 and 2, as well as the plant-derived opioid peptides rubiscolin-5 and rubiscolin-6. All the peptides displayed in vitro antioxidant activity measured with different assays. When incubated with neuronal SH-SY5Y cells, neither endomorphins nor rubiscolins showed any cytotoxicity but were able to protect SH-SY5Y cells in the presence of the neurotoxin 6-hydroxydopamine. The proposed mechanism was different between endomorphins and rubiscolins. Endomorphins acted as anti-apoptotic agents, whereas rubiscolin (and in particular rubiscolin-6) prevented oxidative stress in SH-SY5Y cells. The studied peptides can be promising neuroprotective agents that deserve further exploitation in the treatment of Parkinson’s disease.

Dietary proteins can be an excellent source of bioactive peptides, with several biological activities. Fleury et al. [7] investigated the ability of some dietary proteins and the peptides derived from their in vitro digestion to inhibit the activity of the enzyme dipeptidyl-peptidase IV (DPP-IV). This enzyme is a pharmacological target in type-2 diabetes therapy since it is involved in the degradation of the insulinotropic incretin hormones. They applied both in vivo and in vitro studies to assess the DPP-IV inhibitory potential of selected dietary proteins. In the in vivo studies, they measured the activity of DPP-IV in rat plasma after the consumption of dietary proteins. In the in vitro approach, proteins were firstly digested and then the DPP-IV-inhibitory activity was assessed in a Caco-2 cell models. Reported results showed that vegetable proteins and haemoglobin exhibited the most potent in vivo DPP-IV-inhibitory activity, whereas in the in vitro approach haemoglobin, caseins and whey proteins yielded the highest inhibitory effect. Using peptidomics and in silico analysis, several potential DPP-IV-inhibitory peptides were identified. These results suggest the possible utilization of some dietary proteins in the framework of personalized nutrition for the treatment of type-2 diabetes.

Honisch et al. [8], instead, analysed the anti-tyrosinase activity of some cyclic peptides derived from antamanide. This cyclic peptide has been firstly isolated from the mushroom Amanite phalloides and characterized for several pharmacological activities. Tyrosinase is a ubiquitous enzyme found in numerous organisms, involved in the hydroxylation of mono-phenols and the successive oxidation of di-phenols in the corresponding quinones. The formed quinones are reactive molecules that can form brown pigments. This enzyme is involved in the hyperpigmentation of the skin, for example, in melasma and age spots, as well as in the non-enzymatic browning in fruits and vegetables. Therefore, the authors tested antamanide and some glycine-derived antamanide peptides for their ability to inhibit tyrosinase. Despite the original peptide antamanide not showing any inhibitory activity, the derived cyclic peptides were able to inhibit tyrosinase activity. Finally, by using molecular docking studies the binding mechanism of the inhibitory peptides with the catalytic site of tyrosinase was elucidated.

Finally, two review articles have been published in the Special Issue. One article [9] reviewed the current and future pharmacological applications of vasopressin and its analogues, such as lypressin, desmopressin and terlipressin. The review thoroughly describes the biology of vasopressin, the structure of natural and synthetic analogues and their application in medicine. The second review paper [10] describes the functions and the potential clinical applications of blood neutrophil-derived products, including anti-microbial peptides as well as microvesicles and neutrophil degranulation products. The article begins with an in-depth discussion of the type and role of anti-microbial peptides produced by circulating neutrophils. They move on to describe the actual and future clinical applications of these anti-microbial peptides. Finally, they discuss the clinical applications and future perspectives of other neutrophil-derived products.

In conclusion, we wish to thank the authors for their valuable contribution to this Special Issue, providing their latest research in the field of bioactive peptides and their impact on human health. Although the above-mentioned articles cannot be considered an exhaustive picture of the research in the area of bioactive peptides and health, this Special Issue constitutes an important collection of papers in a growing field of research with substantial implications in the health, disease and food sectors.
